# Lessons learned from the conduct of inpatient clinical trials in a pandemic

**DOI:** 10.1017/cts.2024.483

**Published:** 2024-10-15

**Authors:** Jane A. O’Halloran, Jeremy R. Beitler, Lucy K. Chung, Mamta K. Jain, Akram Khan, Lisa H. Merck, Ahmad Mourad, Minn A. Oh, Shweta Sharma, Tammy Yokum, Seema U. Nayak

**Affiliations:** 1 Washington University School of Medicine, St. Louis, MO, USA; 2 University College Dublin, Dublin, Ireland; 3 Columbia University, New York, NY, USA; 4 Department of Health and Human Services, National Institute of Allergy and Infectious Diseases, National Institute of Health, Bethesda, MD, USA; 5 UT Southwestern Medical Center, Dallas, TX, USA; 6 Oregon Health and Science University, Portland, OR, USA; 7 Department of Emergency Medicine, Virginia Commonwealth University, Richmond, VA, USA; 8 Department of Medicine, Division of Infectious Diseases, Duke University School of Medicine, Durham, NC, USA; 9 Division of Biostatistics and Health Data Science, School of Public Health, University of Minnesota, Minneapolis, MN, USA; 10 Division of Microbiology and Infectious Diseases, National Institute of Allergy and Infectious Diseases, National Institute of Health, Rockville, MD, USA

**Keywords:** ACTIV, COVID-19, clinical trial, inpatient, pandemic preparedness

## Abstract

**Background:**

The COVID-19 pandemic amplified known challenges associated with the conduct of inpatient clinical trials, while also introducing new ones that needed to be addressed.

**Methods:**

Stakeholders based in the United States who participated in the conduct of inpatient therapeutic trials for the treatment of COVID-19 as part of the Accelerating COVID-19 Therapeutic Interventions and Vaccines program identified challenges experienced in the conduct of these trials through a series of meeting to discuss and identify common themes. In addition, innovations developed to address these challenges and other potential solutions that may be utilized in future pandemics were highlighted.

**Results:**

Six thematic challenges including infection control considerations, the interplay between provision of clinical care and research, competing clinical trials, arduous consenting procedures, onerous procedural requirements, and participant recruitment including achieving representation of diverse populations were identified and are discussed here.

**Conclusions:**

Consideration of the lessons learned and recommendation outlined here may allow for more efficient conduct of inpatient clinical trials in future pandemics.

## Introduction

The overall goal of the US National Institutes of Health (NIH) Accelerating COVID-19 Therapeutic Interventions and Vaccines (ACTIV) initiative was to rapidly develop SARS-CoV-2 medical countermeasures to mitigate COVID-19 morbidity and mortality and to accelerate the end of the pandemic. When ACTIV first started, little was known about the pathogenesis of SARS-CoV-2. Despite this limited knowledge, the ACTIV program successfully deployed 11 master protocols to evaluate 37 potential agents for the treatment of COVID-19 and enrolled more than 24,000 participants. More than half of trials evaluated therapies for patients with severe COVID-19 requiring hospitalization. This article describes the challenges faced by those conducting the ACTIV inpatient clinical trials and outlines potential solutions to be deployed in future pandemics.

## Methods

**Figure 1.**
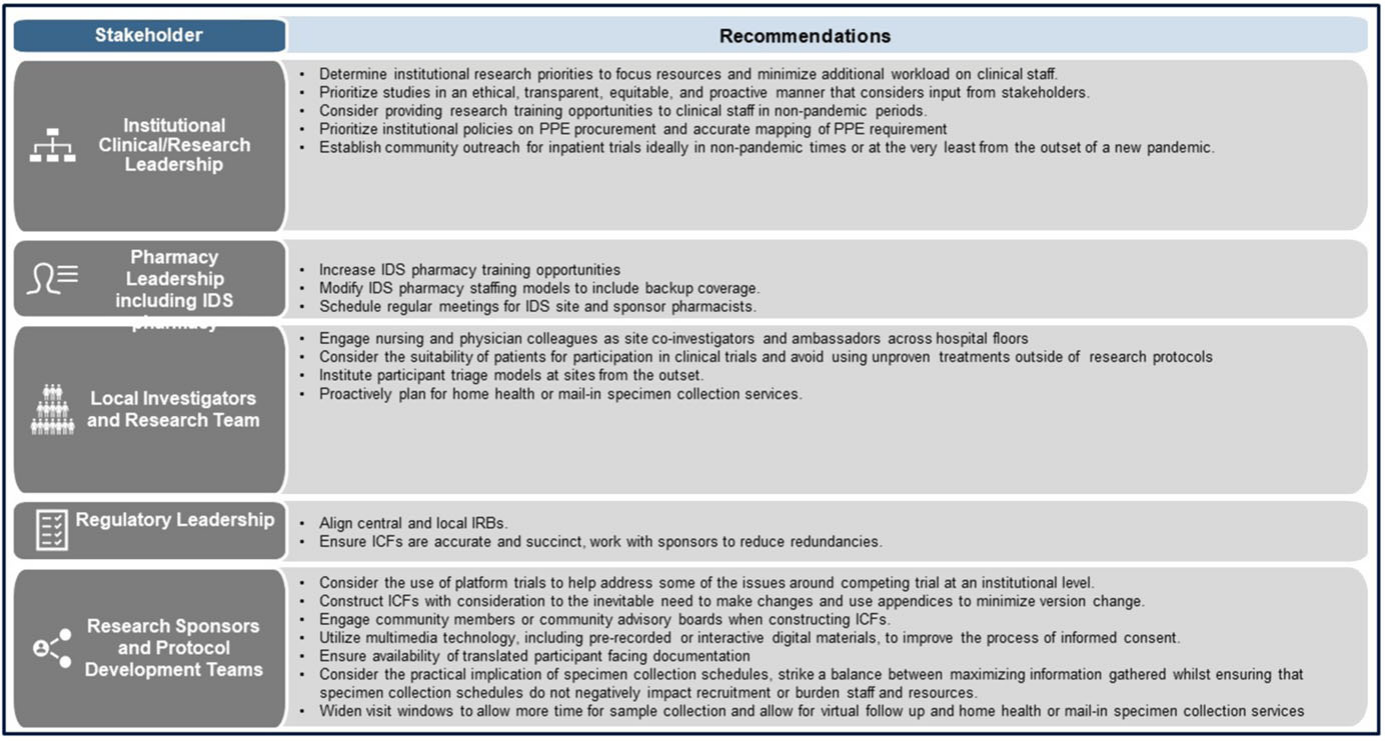
ACTIV inpatient recommended solutions for future pandemic responses. ACTIV = accelerating COVID-19 treatment interventions and vaccines; EUA = emergency use authorization; ICF = informed consent forms; IDS = investigational drug service; IP = investigational product; IRB = institutional review board; PPE = personal protective equipment.

Our writing group consists of stakeholders based in the United States who participated in the conduct of inpatient therapeutic trials for the treatment of COVID-19 as part of the ACTIV program. The group includes clinical investigators from infectious diseases, pulmonary, critical care, and emergency medicine; physicians, nurses, pharmacists, protocol managers, and sponsor representatives. To identify challenges experienced in the conduct of these trials, the group held a series of meeting to discuss and identify common themes. Here we outline six thematic challenges identified by the writing group. These include competing clinical trials, the interplay between provision of clinical care and research, infection control considerations, arduous consenting procedures, onerous procedural requirements, and clinical trial recruitment issues, including representation of diverse populations in the trials. Within these themes, individual challenges were identified and, where possible, precipitating factors discussed. Lessons learned are outlined, and recommendations for the future are proposed (Figure 1).

## Competing clinical trials

### Challenge

Numerous trials at individual sites being led by different investigators.

#### Precipitating factors

From the outset of the pandemic, researchers from a variety of disciplines raced to address the multitude of unknowns surrounding SARS-CoV-2. While these efforts were critical, they occasionally contributed to challenges in the conduct of clinical trials at the site level. Problems occurred when research teams with different principal investigators (PIs) and potentially from different departments worked in silos, while competing for the same limited pool of potential participants. Institutions and researchers faced the challenge of effectively and efficiently determining which competing trials had the best chance of benefiting current and future patients with COVID-19 disease and establishing a transparent and ethical process to recruit participants while maintaining research integrity [1]. While this issue is not new [2], it was exacerbated during COVID-19 pandemic.

#### Lessons learned

Some institutions established clinical trial review committees to evaluate the scientific merit of proposed research, the patient/institutional burden, safety, and other factors, and in some instances provide a priority ranking for individual studies within their institutions [1,3]. A survey of 60 clinical and translational science award (CTSA) centers showed that 87% of the CTSAs established a feasibility committee to review COVID-19 research, and 23% set up prioritization committees to review study feasibility, competing trials, and process management [4].

#### Recommendations

When establishing prioritization of potential trials for future pandemics, it is important to understand how this will affect relevant stakeholders. Investigator considerations, such as the impact of prioritization on their research program, and sponsor considerations, such as the impact of local prioritization on study completion timelines, should be taken into account. Institutional prioritization of studies, if done, should be conducted in an ethical, transparent, equitable, and proactive manner that considers input from all stakeholders.

An alternative strategy in a future pandemic is to consider national prioritization of trials. While this approach was taken in some countries during the COVID-19 pandemic, it too raises inherent practical challenges as well as concerns that research ingenuity and innovation could be stifled.

### Challenge

Participant approach and recruitment in the setting of multiple trials.

#### Precipitating factors

ACTIV inpatient clinical studies simultaneously executed multiple trials of experimental therapies with partial or complete overlap of inclusion/exclusion criteria within each participating institution. Additionally, most institutions had other non-ACTIV COVID-19 trials open and recruiting across emergency departments and inpatient units. This raised ethical issues in participant selection and informed consent [4,5]. At some sites, it was difficult to offer patients the choice of all potential experimental interventions for COVID-19, as teams were concerned that the presentation of multiple protocols to critically ill patients or their family members would be overwhelming. Additionally, the need for researcher to wear masks and eye coverings and the use of respiratory devices by patients added additional communication challenges. At times, multiple teams approached the same patient for different trials, causing confusion and reluctance on the part of patients to participate in any study. On some occasions, it also led to patient frustration, especially if they had already expressed their desire not to be involved in research studies.

#### Lessons learned

Some institutions, including those participating in ACTIV inpatient studies, struck a balance between autonomy and the ability to enroll patients in ongoing studies by prioritizing trials and mandating communication between the various research teams [1,3]. At some sites, this took the form of daily triage meetings between research teams, either in person or through a secure group chat, to review patients and discuss the most appropriate trials for each patient. Patients could be offered a single study if they met the inclusion criteria. If the patient declined the study, other teams would only contact them if they had expressed interest in future research opportunities.

#### Recommendations

In future pandemics, instituting similar models of trial/participant triage at sites from the outset should be encouraged. At a sponsor level, use of a platform trial design such as ACTIV can help address some of the issues around competing trials at an institutional level. This helps reduce the need for several separate trial teams, while continuing to ensure that research on multiple agents occurs. From this perspective, sponsors should be encouraged to consider such strategies in future pandemic situations.

## Interplay between clinical care and research conduct

### Challenge

Increased burden on clinical staff.

#### Precipitating factors

Due to the shortage of personal protective equipment (PPE) and desire to limit staff exposure, research-specific tasks, such as approaching patients to assess interest in research participation, collecting research specimens, and administering study medications were often completed by the available clinical staff. Because infection control precautions in many hospitals precluded taking paper consent forms out of rooms, some institutions required clinical staff to act as independent witnesses to the consent process, increasing clinical team time demands. Some protocols mandated training for drug administration, placing additional burden on the clinical teams.

#### Lessons learned

Pragmatic protocols that considered the potential implication of study activities not only on the participants but also on the clinical care team and infrastructure, were essential.

#### Recommendation

Institutional clinical and research leadership need to determine research priorities and to focus resources on awareness and educational campaigns for non-research staff to prioritize protocols that affect pandemic care. In addition, institutions should consider providing research training opportunities to clinical staff in non-pandemic periods to foster interest in clinical trials and an understanding of their vital role in addressing knowledge gaps. This would contribute to an environment where clinical teams are prepared to incorporate research into clinical care during pandemic situations.

### Challenge

Communication barriers between clinical care and research teams.

#### Precipitating factors

At certain sites, research staff who enrolled patients, collected samples, and administered study medication had limited ability to communicate with the front-line clinical staff. Research teams could not document notes in the electronic medical records (EMR) in keeping with pre-pandemic local guidelines. Real-time email contact with clinical staff was not feasible due to demands on their time and the ability to contact clerical staff to obtain the patient’s bedside phone number was also a challenge due to staff availability. As a result, research teams had difficulty accessing the clinical infrastructure to notify the clinical team when a patient was starting or stopping study medication, needed research laboratories drawn, or to obtain real-time information on adverse events. Coordinating research procedures with clinical care was essential given the risk and costs of repeated in-person contact with the patient, preservation of PPE, avoidance of extra-blood draws, and prevention of adverse events related to drug-drug interactions or protocol noncompliance. In some instances, the lack of interaction across the clinical and research teams was due to separate employment (university versus hospital versus temporary staffing) as well as access to hospital systems.

#### Lessons learned

Trial teams explored strategies to overcome communication challenges between research and clinical staff, which in turn reduced the burden of trial-related duties placed on clinical staff. These strategies included efforts to promote awareness of clinical trial procedures among clinical staff working in units where research participants were enrolled. The use of EMR messaging applications or other HIPAA-compliant communication methods (such as Microsoft Teams, SPOK, and Secure Chat) significantly improved communications where they were implemented between research staff and the clinical team. Some institution included a member of the research team on clinical rounds, which helped reduce the burden on bedside staff and improved safety. For sites with pre-pandemic inpatient clinical trials activities, existing relationships from pre-pandemic trials greatly facilitated communication between research and clinical staff during pandemic trials.

#### Recommendations

Overall, a collaborative environment is required. Engaging nursing and physician colleagues as site co-investigators and ambassadors across hospital floors (from the Emergency Room through the intensive care units [ICUs]) increases trust, understanding of the protocols, and accountability for protocol adherence. Sponsors may be able to contribute to the collaborative environment by supporting inpatient trials networks during non-pandemic times so that lines of communication between research teams, clinical leadership, and bedside clinical staff are established and to maintain cross-disciplinary relationships longitudinally.

### Challenge

Overlap between care and research.

#### Precipitating factors

Early in the pandemic, providers used therapies with unproven benefit against COVID-19 disease (e.g., ivermectin and hydroxychloroquine) given the absence of evidence for any specific therapy and a (well-intended) desire “to do something” [6]. Ultimately this practice undermined clinical trial testing of novel therapeutics for SARS-CoV-2 and contributed to reluctance for some patients to take part in clinical trials. In addition, expanded access programs and emergency use authorizations were used as mechanisms for patients to access treatments. However, lack of knowledge around these mechanisms by both providers and patients meant that they were frequently confused with clinical trials, particularly where written informed consent was required, and this also impacted patients’ interest in participating in clinical trials. Over time, evidence for specific drugs was obtained; however, access to these drugs was not widely available in certain regions due to limited supplies. Participating in clinical trials sometimes offered access to standard of care (SOC) treatment, such as remdesivir, in addition to experimental therapy, which helped reduce disparities but contributed to patient confusion regarding drug approval status.

#### Lessons learned

We have demonstrated that large, timely, pragmatic clinical trials can be conducted rigorously during a pandemic and provide invaluable evidence to guide emergency authorizations and full drug approvals by regulators, as well as therapeutic decision-making by clinicians. In contrast, observational and anecdotal data can be misleading and consume scarce resources potentially without benefit to individual patients (lack of efficacy) or the population health (limited knowledge gained). Study teams were able to help educate providers on available SOC and how study participation could reduce disparities (when SOC was limited).

#### Recommendations

In future pandemics in which effective treatments are unknown, clinical teams should be encouraged to consider the suitability of their patients for participation in clinical trials and to avoid using unproven treatments outside of trial protocols. Priority should be placed on conducting randomized trials and avoiding non-evidence-based emergency use authorizations (EUAs).

### Challenge

Complex requirements for investigational product.

#### Precipitating factors

Lack of robust inpatient investigational drug pharmacy infrastructure, particularly with after-hours capacity, was a key factor driving issues with management of investigational product (IP) and impacted the enrollment and conduct of trials. In particular, pharmacy support for evening and weekend enrollments was critically lacking at some sites. Due to workload and staffing issues, many clinical and staff pharmacists were unable to help with IP needs. This led to a lack of pharmacists trained in the Good Clinical Practice (GCP) and protocol-specific preparation and ultimately limited study recruitment.

In addition, communicating with clinical staff to coordinate bedside drug delivery was time consuming. Drug administration, particularly for intravenous preparations, was often complicated by the need to coordinate with other continuous infusion medications due to intravenous line access or pump and agent compatibility.

#### Lessons learned

The lack of robust investigational pharmacy services became apparent during the pandemic. Temporary work-arounds were developed, such as developing an on-call system for research pharmacists whenever a new enrollment occurred. Nevertheless, expansion in staffing schedules ultimately was required.

#### Recommendations

To support enrollment and dispensing of study products on weekends, research pharmacies can modify their staffing model to include backup coverage. Non-research pharmacists would need to be trained in GCP and the protocol, which requires time, effort, and commitment from pharmacists and study staff, support from the pharmacy and hospital leadership, buy-in from current investigational drug pharmacy personnel, and sponsor support and resourcing.

Investigational Drug Service (IDS) pharmacy is a niche field with unique concerns, and regularly scheduled meetings for study pharmacists across sites participating in the same clinical trial together with pharmacists at the sponsor level, could improve efficiencies and further support clinical pharmacists not regularly working in research. Site pharmacists can discuss specific challenges, concerns, and questions with peers to identify potential solutions. A sponsor-level pharmacist is extremely important, as many issues specific to the trial are understood only by pharmacist colleagues who are experienced and educated in the specialized area of clinical investigational drug trials.

## Infection control considerations

### Challenge

PPE shortages.

#### Precipitating factors

PPE shortages resulted in dire conditions, with institutions scrambling to obtain PPE for essential clinical care. This resulted in limited availability of PPE for all other activities, including research. In addition to clinical research staff, limited availability of PPE also affected laboratory and pharmacy staff. Pharmacy personnel who prepare sterile intravenous admixtures must wear sterile PPE to minimize the risk of microbial contamination. Due to demand and lack of knowledge, the allocation of PPE did not always include prioritization of PPE to pharmacies. This forced the staff who compounded sterile products to reuse masks, hair bonnets, shoe covers, and gowns, increasing the risk of microbial contamination, resulting in concerns about safety and infection control. Shortages also limited the ability of research laboratory personnel to conduct both COVID-19 and non-COVID-19-related research.

The factors behind this shortage varied from country to country, but main themes included: (1) a rapid increase in demand for PPE from healthcare systems and the general public; (2) use of just-in-time ordering systems driven by a PPE budgeting model, where PPE is an expense incurred by healthcare systems; (3) a breakdown in the global supply chain of PPE, which arose from dependence on production within limited geographic regions, compounded by workforce illness and isolation restrictions, and complicated by export bans; as well as (4) failure of governments to maintain adequate national PPE stockpiles and delays in responding to this shortfall through increased production [7].

#### Lessons learned

Efficient trial conduct requires access to appropriate PPE in a timely manner for all key stakeholders, including those who many not be patient facing such as laboratory and pharmacy staff. PPE shortages led to significant research to determine the safety and efficacy of reusing certain PPE elements, such as face masks and goggles. This data can inform the use of PPE in future pandemic situations and can be incorporated into pandemic preparedness guidelines [8] to encourage appropriate behavior from the outset.

#### Recommendations

Factors such as institutional policies on PPE procurement and accurate mapping of PPE requirements, as well as government policies on maintaining and distributing PPE stockpiles, should be prioritized for future responses.

## Arduous consenting procedures

The processes and ethics of informed consent for clinical research have been debated and discussed well before the onset of the COVID-19 pandemic. Conducting clinical trials of COVID-19 therapeutics during the pandemic highlighted old and new challenges with informed consent.

### Challenge

Level of literacy required and length of informed consent forms (ICFs).

#### Precipitating factors

As outlined in a 2021 commentary, most scientific societies and regulatory bodies recommend writing ICFs at a level of comprehension equivalent to an eighth-grade education [9,10]. However, several studies have shown that half of adults in the USA cannot read at the eighth-grade level, and almost one-third only have “basic sight vocabulary and can read short texts on familiar topics to locate a single piece of information” [11,12]. Additionally, one article in 2004 evaluating consent forms for oncology trials suggested that these forms should be limited to ≤ 1,250 words [13]. In retrospect, during the pandemic numerous consent forms were written for higher levels of comprehension than the eighth-grade level [14] and were almost 7,000 words long – over five times the recommended length suggested.

#### Lessons learned

Although the importance of balanced informed consents is long established, its significance was magnified during the pandemic as a result of enhanced communication challenges. In addition to optimizing ICFs, the introduction of communication aids such as flip-books (illustrated aids describing the trial) improved recruitment and patient experience.

#### Recommendations

Ensure that informed consent forms are accurate and succinct, and where necessary, work with regulatory bodies and institutions to reduce redundancies and streamline mandated statements. Engage community members or community advisory boards when constructing these forms to help improve comprehension and understanding.

### Challenge

Trials needed ICFs in multiple languages [15].

#### Precipitating factors

Providing accurate consent forms in multiple languages required appropriate translation services which were costly and time consuming. Additionally, the availability of translation services varied at the enrollment sites, which may have prevented the enrollment of some patients.

#### Lessons learned

Some trial networks queried sites for languages they were likely to encounter and preplanned central translation of ICFs and other patient-facing materials for those languages. Short-form ICFs were sometimes provided to sites, while long-form translation was pending for certain languages.

#### Recommendations

Providing comparable information to both English and non-English speakers during the consent process facilities health equity. Ensuring availability of translated participant-facing documentation needs to be prioritized from the outset and appropriately resourced to ensure that underrepresented populations are not further disadvantaged and can receive the same information via the same materials when deciding whether to participate in clinical trials.

### Challenge

Rapidly evolving ICFs.

#### Precipitating factors

Although platform trials allowed the study of multiple therapeutic agents simultaneously, adding and removing study arms at intervals throughout the trial required frequent updates to the ICFs, which necessitated prompt review by local and central institutional review boards or their equivalent, as well as retraining of study personnel.

#### Lessons learned

Although updates to ICFs were inevitable during the pandemic, the pace at which they were amended was sometimes overwhelming for study teams. However, introducing strategies such as the use of appendixes to indicate interventions being studied at a given time or site was a helpful strategy that avoided the need for modification of the main ICF.

#### Recommendations

Sponsors and investigators should thoughtfully construct ICFs with an eye to the inevitable need to make changes, and use carefully designed appendices to minimize version change for the main ICF document where possible.

### Challenge

Lack of coordination between central/single and local Institutional Review Boards (IRBs).

#### Precipitating factors

Central IRBs allow for the streamlining of several processes, including the approval and distribution of accurate ICFs to multiple sites. However, differences in the requirements of central and local IRBs caused delays in the approval of site-specific ICFs. Sites would then have a lag between receiving the central IRB-approved consent form and the institution allowing its use, leading to protocol deviations.

#### Lessons learned

Local IRB requirements that do not take into account Central IRB processes lead to timeline delays and protocol deviations. Government regulations mandating central IRBs have had the unintended effect at some sites of producing duplicative review and work rather than streamlined network-wide approvals.

#### Recommendations

Sponsors and governments should strongly encourage central and local institutional review boards to align to decrease redundancy in approval processes.

### Challenge

Restrictions on visitors and removal of items from the participant’s bedside.

#### Precipitating factors

Toward the beginning of the pandemic, the degree to which fomites were responsible for SARS-CoV-2 transmission was unknown. Although this risk was ultimately determined to be very low, early concerns resulted in policies being implemented to prevent/minimize the removal of items from the patient care area. From a research perspective, these restrictions significantly impacted the consent process, with onerous workarounds such as the use of impartial witnesses increasing the complexity of the consenting process. Site personnel had to approach and recruit patients while wearing PPE, which made consenting patients who had hearing impairment or who were on loud high-flow high-humidity nasal cannula systems difficult. Additionally, given visitor restrictions, patient families, or surrogate decision-makers had to be contacted by phone, often from inside the patient room – which was also made difficult by the need for research personnel to wear PPE.

#### Lessons learned

The rollout of electronic consenting (eConsents) during the pandemic was important in addressing infection control issues impacting consenting. Tablets and other devices improved the in-person consenting process in some settings and when used for electronic consent and multimedia resources they could be removed from patient rooms and sanitized. Many trial sites did not have local systems for remote eConsent with electronic signature in place, presenting a major barrier to participant enrollment through remote surrogate consent.

#### Recommendation

Utilize multimedia technology, including pre-recorded or interactive digital materials, to improve the process of informed consent and address infection control concerns by accepting electronic signatures from portable electronic devices. Until mechanisms for remote eConsent are ubiquitous, sponsors should consider making the requisite tools available to all network sites to facilitate participant recruitment when the legally authorized representative is not available for in-person written informed consent.

## Onerous trial procedures

### Challenge

Impractical specimen collection schedules.

#### Precipitating factors

Due to the lack of understanding of the pathophysiology of SARS-CoV-2 at the outset, some protocols included intensive specimen collection schedules. These sampling schedules increased pressure on clinical and study staff and increased consumable and PPE use at a time when both were in short supply. In some circumstances, such as with protocols that included daily nasopharyngeal swabs for example, aggressive collection schedules disincentivized participation.

#### Lessons learned

Having observed the challenges outlined above, many protocol teams streamlined their specimen collection schedule and prioritized the use of standard of care laboratory results where possible. This approach relieved pressure on both the participants and the clinical and research teams.

#### Recommendations

Protocol development teams and sponsors need to consider the practical implications of specimen collection schedules and strike a balance between maximizing information that can be gathered from a study whilst ensuring that aggressive specimen collection schedules do not negatively impact recruitment or burden staff and resources. This is particularly important when collecting specimens for unspecified future secondary research. Involving community representatives in the development of such collection schedules is essential.

### Challenge

Specimen collection after hospital discharge.

#### Precipitating factors

Specimen collection post discharge was a significant challenge for inpatient COVID-19 trials. Enrolling sites were hospitals and many sites had infection control-related policies that prevented participants from returning for in-person visits due to quarantine requirements and lacked outpatient facilities authorized to see participants. Furthermore, for participants who lived far from the enrollment sites, travel back to the trial site for follow-up was not feasible, especially for early visits that were a few days apart. For this reason, participants frequently refused to participate in protocols that included mandatory post discharge study visits.

#### Lessons learned

For some studies, sponsors contracted with home-health phlebotomy service to provide access to sites. This central approach eliminated the need for sites to contract with companies themselves. These services were not available in all locations, and in such circumstances, some sites were able to contract with their own local providers. Alternatively, mail-in home collection kits were used for some sample types.

#### Recommendations

Where post discharge specimens are deemed to be scientifically important, study teams should proactively plan for home health services or mail-in collection services from the outset and include these in the study design and budget. To assist with in-home specimen collection, where feasible and clinically appropriate, protocol teams can widen visit windows to allow more time for sample collection.

### Challenge

Maintaining follow-up.

#### Precipitating factors

Maintaining follow-up after hospital discharge is always challenging, and this challenge was exasperated during the pandemic. Some clinical trials had extensive follow-up requirements after the patient was discharged from the hospital, which was problematic, particularly if participants still required quarantine. In addition to the site challenges outline above for in-person visits, participants often struggled to get transportation while still in quarantine.

As participants recovered, their focus shifted back to jobs, childcare, and other obligations. Reaching participants when they were not home, i.e., during admission at another facility (hospital or rehabilitation center) was also challenging and frequently time consuming. Once contact was made, participants did not recall specific dates or have clinical data available from the time spent in the other hospital or rehab facility. Obtaining medical records from other facilities required additional paperwork and time.

Due to hospital policies during the pandemic, face-to-face contact with participants was limited, affecting the ability of research staff to develop relationships with participants and possibly compromising their ability to maintain contact after discharge. Research staff turnover also impacted follow-up data collection.

#### Lessons learned

Protocols that were developed or adapted to incorporate virtual study visits, with or without home-based specimen collection, were essential for the successful retention of participants in clinical trials during the pandemic and many studies including those in the ACTIV network made this transition successfully.

#### Recommendations

Where feasible, trials should be designed to include telehealth follow-up from the outset. Such plans should account for differing access to telehealth resources (especially video) and provide alternative plans for follow-up for those without access to required technology. This can reduce some of the burden for participants to take time off from work, find transportation or interfere with other activities, and make trial participation more appealing. Maintaining trusting relationships with trial participants and ensuring adequately trained staff are critical to improving trial attrition [16]. Future trials should carefully consider the follow-up time necessary to collect data for the primary outcome and the value of extended follow-up.

## Clinical trial recruitment

### Challenge

Underrepresented populations.

#### Precipitating factors

Clinical trial cohorts should reflect the demographics of populations impacted by the study disease. Representation in clinical trials: (1) ensures generalizability of trial results; (2) facilitates community acceptance of trial results; and (3) promotes health equity as a means of providing access to potentially beneficial treatments [17].

Racial and ethnic minorities are often underrepresented in clinical trials, particularly in phase II and III trials, where the prospect of direct benefit is greatest [18,19]. Pregnant people and those at the extremes of age are sometimes excluded outright from trial participation, leaving significant gaps in data for these populations. Contributors to underrepresentation include structural factors, trial design, mistrust of the medical research community, participant time burden, variable access to information, and stigma [20].

Inpatient trials can only recruit the population at participating trial sites. Inpatient trial sites are generally academic medical centers, although the degree to which such hospitals serve populations representative of the surrounding geographic area is quite variable [21]. Given the time pressures of the pandemic, preexisting trial networks were often redeployed for COVID-19 trials. Representation of pandemic study populations thus hinged partly on whether preexisting trial sites collectively recruited from diverse populations.

#### Lessons learned

In a novel global pandemic without established effective therapeutics, representation is of critical importance, and failure to achieve a representative trial population risks cascading effects on health disparities. During the pandemic, numerous strategies were deployed to improve representativeness such as community outreach, attempts to minimize barriers to study enrollment, and adding new sites to existing networks – including sites in rural and community settings outside the traditional academic medical center structure.

#### Recommendations

Include trial sites that collectively care for populations reflective of the diversity of regional populations and the country. Establish community outreach for inpatient trials ideally in non-pandemic times or at the very least from the outset of a new pandemic to enhance representation by facilitating openness to patients and families when individuals from the community are hospitalized. Building community-representative research teams, integrating cross-cultural perspectives in training research staff, and developing recruitment materials may also improve representation [22]. Accurate surveillance data following the course of epidemics and pandemics are essential to ensure that the populations most affected by the pathogen of concern are well represented in clinical trials [23]. Finally, minimizing barriers to enrollment and considering social determinants of health that disproportionately affect underrepresented populations should be taken into account when designing clinical trials.

### Challenge

Slow enrollment.

#### Precipitating factors

Although many of the initial inpatient clinical trials recruited at phenomenal speed, as time went on, it became increasingly difficult to rapidly recruit inpatient trials. On a positive note, some of this reflected the improving clinical epidemiology. However, other factors such as skepticism toward research, competing trials, bureaucratic delays in opening trial sites, and inability to recruit research staff also contributed significantly.

#### Lessons learned

In addition to the lessons outlined above for competing trials and recruitment of underrepresented populations, in many incidences successful recruitment hinged on having a large global network of sites available for participation. However, establishing and developing these networks took considerable time, effort, and resources.

#### Recommendations

Maintaining established global networks through warm base funding and an ongoing research portfolio in non-pandemic times will ensure that trial initiation in a future pandemic will be faster and that the skills and expertise needed to carry out these trials will be preserved.

## Conclusions

The COVID-19 pandemic raised so many challenges across the domains of medicine. However, valuable lessons were learned for the stakeholders involved in conducting clinical research in all research settings, particularly in the inpatient clinical trials arena. While we believe that the American experience underlying this report is representative of the challenges experienced by trialists worldwide, we observe that every site is unique and likely had to overcome site-specific challenges as well. Our hope is that implementation of our recommendations will address many of the common barriers and allow greater focus on site-specific problems. Although some of the recommendations pertain to what should be done in the event of a future pandemic, it is important to note that much of the ground work can be initiated now to ensure readiness in the future.
